# Enzymatic Hydrolysis of Gluten in Beer: Effects of Enzyme Application on Different Brewing Stages on Beer Quality Parameters and Gluten Content

**DOI:** 10.3390/foods14142519

**Published:** 2025-07-18

**Authors:** Carolina Pedroso Partichelli, Vitor Manfroi, Rafael C. Rodrigues

**Affiliations:** 1 Biotechnology, Bioprocess and Biocatalysis Group, Institute of Food Science and Technology, Federal University of Rio Grande do Sul, Avenida Bento Gonçalves, 9500, P.O. Box 15090, Porto Alegre ZC 91501-970, RS, Brazil; carolpartichelli@gmail.com; 2 Beverages Technology Lab, Institute of Food Science and Technology, Federal University of Rio Grande do Sul, Avenida Bento Gonçalves, 9500, P.O. Box 15090, Porto Alegre ZC 91501-970, RS, Brazil; manfroi@ufrgs.br

**Keywords:** beer, gluten, AN-PEP enzyme, foam stability

## Abstract

A rising demand for low-gluten beer fuels research into enzymatic solutions. This study optimized *Aspergillus niger* prolyl endopeptidase (AN-PEP) application timing during brewing to reduce gluten while preserving physicochemical quality. Ale-type beers were produced with AN-PEP (2% *v*/*v*) added at mashing, boiling, post-boiling, or post-fermentation, plus a control. Three mashing profiles (Mash A, B, C) were also tested. Gluten was quantified by R5 ELISA (LOQ > 270 mg/L). Color, bitterness, ABV, and foam stability were assessed. Statistical analysis involved ANOVA and Tukey’s HSD (*p* < 0.05). Enzyme activity and thermal inactivation were also evaluated. Initial gluten levels consistently exceeded LOQ. Significant gluten reduction occurred only post-fermentation. Mashing, boiling, and post-boiling additions effectively lowered gluten to below 20 mg/L. Post-fermentation addition resulted in significantly higher residual gluten (136.5 mg/L). Different mashing profiles (A, B, C) with early enzyme addition achieved similar low-gluten levels. AN-PEP showed optimal activity at 60–65 °C, inactivating rapidly at 100 °C. Physicochemical attributes (color, extract, bitterness, ABV) were largely unaffected. However, foam stability was significantly compromised by mashing and post-fermentation additions, while preserved with boiling and post-boiling additions. AN-PEP effectively produces low-gluten beers. Enzyme addition timing is critical: while mashing, boiling, or post-boiling additions reduce gluten to regulatory levels, only the beginning of boiling or post-boiling additions maintain desirable foam stability. These findings offer practical strategies for optimizing low-gluten beer production.

## 1. Introduction

The increasing prevalence of gluten-related disorders, such as celiac disease and non-celiac gluten sensitivity, has driven the development of safe and suitable foods for this population [1,2,3]. Among the products that have garnered interest in this context is beer, traditionally brewed with cereals like barley and wheat, which are natural sources of gluten. Although not nutritionally essential, beer plays a significant role in food culture and social interaction, making the search for gluten-free or low-gluten versions that preserve their sensory characteristics particularly relevant [4,5].

The production of gluten-free beers involves significant technological challenges. Substituting traditional cereals with naturally gluten-free grains, such as corn rice or pseudocereals, can compromise the sensory composition of the beverage, leading to products with reduced aroma complexity, foam instability, and decreased body [6]. To overcome these limitations, technological alternatives have been studied, including the use of exogenous proteolytic enzymes capable of hydrolyzing the toxic fractions of gluten without substantially altering the beer matrix [7]. Accurate detection and quantification of gluten are crucial for ensuring the safety of gluten-free diets and adhering to regulatory requirements for “gluten-free” (<20 mg/kg) and “low-gluten” (20–100 mg/kg) labeling, as defined by international standards like Codex Alimentarius [8]. This is particularly challenging in fermented foods like beer, where processing can partially hydrolyze gluten proteins, potentially compromising the accuracy of traditional detection methods [9]. Therefore, selecting an appropriate technique for gluten quantification is crucial.

Current analytical methods for gluten detection offer varying strengths and limitations. Immunological techniques, particularly Enzyme-Linked Immunosorbent Assays (ELISAs), are widely used due to their speed and ease. The sandwich ELISA is recommended for intact gluten but is limited in fermented or hydrolyzed foods. The competitive ELISA, using the R5 antibody, is more suitable for fragmented gluten peptides found in fermented products, as it recognizes single epitopes that may retain immunoreactivity [10,11]. Liquid chromatography coupled to mass spectrometry (LC-MS) offers high specificity and sensitivity, capable of detecting gluten at very low levels and identifying specific peptides, which is valuable when ELISA methods are insufficient [12,13]. While powerful, LC-MS requires specialized equipment and expertise [9]. Molecular methods based on Polymerase Chain Reaction (PCR) target DNA sequences, offering high specificity, but are generally not suitable for highly processed or fermented foods due to DNA fragmentation [14].

While *Aspergillus niger* prolyl endopeptidase (AN-PEP) is widely recognized and utilized, other enzymes have also shown promise in gluten degradation. For instance, proteases from *Aspergillus oryzae* have been investigated for their ability to hydrolyze gluten proteins, contributing to both gluten reduction and improved beer stability [15]. Similarly, studies have explored the use of various bacterial proteases, such as those from the *Bacillus* species, which can offer different pH and temperature optima, potentially allowing for application at various stages of the brewing process [16]. Some research also focuses on combinations of enzymes or novel enzymes from other microbial sources to achieve more comprehensive gluten hydrolysis while preserving beer quality attributes [7].

Prolyl endopeptidase (PEP), especially that derived from *A. niger*, has emerged as a promising tool in this scenario. This enzyme exhibits specificity for proline residues present in the immunogenic sequences of prolamins, which are primarily responsible for adverse reactions in celiac individuals [17]. When applied during the brewing process, PEP can promote the degradation of these proteins, reducing the gluten content of the final beverage to levels acceptable for “low-gluten” labeling, as defined by international standards [18].

Recent studies demonstrate that the enzyme effectiveness depends on factors such as the concentration used, wort composition, and processing conditions, including pH and temperature. In addition to gluten reduction, it is fundamental to ensure that it requires a careful experimental approach for process optimization [7,19].

Although enzymatic gluten-free beer was already studied, the reports published in the literature did not investigate how the application of the enzyme in different brewing steps affect the gluten concentration and physicochemical attributes of the beer. Therefore, the central hypothesis of this study is that optimizing the AN-PEP addition point during the brewing process of high-gravity beer can effectively reduce gluten content to ”low-gluten” levels while preserving critical quality characteristics. This work aims to precisely determine the optimal application strategies for AN-PEP, evaluating its effect on gluten degradation kinetics, foam stability, color, bitterness, and alcohol content, thereby providing practical insights for brewers seeking to reconcile food safety with sensory quality in low-gluten beer production.

## 2. Materials and Methods

Pilsen type malted barley grains (Agraria brand) were purchased in a local market and used exclusively for beer production. Pilsen type malt extract (dry) was also purchased in a local market and utilized for enzymatic activity determination tests. Amarillo hops (T90 pellets, aroma variety) were used during the boiling stage to impart the desired bitterness and aroma profile to the beer. These were supplied by Yakima Chief Hops (United States). The yeast employed was *Saccharomyces cerevisiae*, specifically the SafAle^®^ US-05 strain, an active dry yeast provided by Fermentis (United States). The commercially available AN-PEP enzyme preparation was acquired from DSM. For gluten analysis, the RIDASCREEN^®^ Gliadin Competitive assay kit (Art. No. R7021) was purchased from R-Biopharm. This kit utilizes the Enzyme-Linked Immunosorbent Assay (ELISA) method to quantify gliadin. All other reagents, solvents, and chemicals used were of analytical grade.

### 2.1. Determination of Enzymatic Activity

For the determination of enzymatic activity, the commercial preparation Brewers Clarex^®^ (DSM, Maastricht, TheNetherlands), which contains prolyl endopeptidase (AN-PEP) derived from *Aspergillus niger*, was utilized as the unpurified enzyme solution. The enzyme activity was assessed using a substrate prepared with Pilsen type malt extract, as described below. The method was adopted from Pessato et al. [16,20], using azocasein at 1 mg/mL as a substrate and different enzyme dilutions in a total volume of 1 mL. The reaction was conducted in water bath at 40 °C for 10 min and stopped by adding 1 mL of 16% trichloroacetic acid (TCA). The samples were centrifuged for 10 min at 10,000× *g*, and the supernatant was used for spectrophotometric reading at 280 nm. The blank was prepared similarly, but with the initial addition of TCA. Enzymatic activity was expressed in units (U), defined as the amount of enzyme required to increase the absorbance at 280 nm by 1 µg of tyrosine per minute under assay conditions. An L-tyrosine (Sigma) standard curve was constructed for activity quantification.

### 2.2. Beer Production

Beer production for this study comprised the following main stages: malt milling, mashing, wort filtration, boiling, fermentation, and maturation. For all the experiments, a volume of 400 mL of wort per batch was utilized. The brewing was conducted to produce an original gravity of 15 °Brix (OG 1.061), in order to prepare high gravity beers.

Initially, the malt was milled to optimize sugar extraction and wort permeability. The resulting material was then homogenized in water (100 g of malt and 500 mL of water), previously heated to 50 °C, and maintained for 30 min. The temperature ramp was then raised to 65 °C, maintained for 1 h. Subsequently, the temperature ramp was increased to 75 °C, held for 10 min for enzyme inactivation and starch conversion cessation.

The resulting wort was separated from the grain solids by filtration and washed with 250 mL of water at 80 °C. The liquid phase (wort) was transferred to a boiling kettle, where it was maintained at 100 °C for 60 min. During boiling, 1 g of hop was added at the beginning of boiling (60 min before the end of boiling) for alpha-acid isomerization and aroma development.

After boiling, the wort was rapidly cooled to 20 °C. *Saccharomyces cerevisiae* yeast was then inoculated into the cooled wort (0.32 g to 400 mL of wort), and the fermentation process was conducted at 20 °C for 7 days.

Following primary fermentation, the beer was transferred to amber bottles and maintained at 4 °C for 20 days for maturation. This stage promoted natural carbonation and beer conditioning, contributing to the enhancement of the sensory profile and the removal of undesirable volatile compounds. Clarification occurred naturally during maturation. A control batch, without any addition of the AN-PEP enzyme, was produced strictly following this standard protocol.

For the experimental beers, the AN-PEP enzyme was added at a concentration of 2% (*v*/*v*) relative to the 500 mL batch volume. Experiments were divided into two series: evaluation of the enzyme addition point in the process and evaluation of the impact of different mashing ramps. These experimental conditions, including enzyme addition points, mashing profiles, and estimated enzyme exposure conditions, are schematically summarized in Table 1.

### 2.3. Quantitative Gluten Analysis

Gluten content in beer samples was quantified using the R-Biopharm (Darmstadt, Germany) competitive R5 ELISA test kit, Art. No. R7021, following the manufacturer’s standardized method. This enzyme immunoassay employs the R5 monoclonal antibody, recognized for the detection of toxic prolamins from gluten-containing cereals.

The procedure included protein extraction from sample using a “fish gelatin ethanol” solution (60% ethanol solution, containing 10% liquid fish gelatin). The competitive reaction occurred with the R5 antibody conjugated to an enzyme in wells of plates pre-coated with gliadin. Colorimetric quantification was performed after the addition of the enzymatic substrate, with absorbance reading in a spectrophotometer at a wavelength of 250 nm.

Calibration curves were generated with gliadin standards at concentrations of 0, 10, 30, 90, and 270 ng/mL. Gluten concentrations in the samples were determined by interpolation from the calibration curves, with results expressed in mg/L (Limit of Detection (LOD) < 10 mg/L; Limit of Quantification (LOQ) > 270 mg/L). All analyses were conducted in duplicate.

### 2.4. Qualitative Beer Analyses

#### 2.4.1. Colorimetry and Bitterness Analysis

Beer color was determined by UV-Vis spectrophotometry using a Shimadzu spectrophotometer (UV-Vis model UV-1900i, Shimadzu, Kyoto, Japan). The analysis was conducted according to the ASBC Methods of Analysis Color (Beer-10). The protocol was developed by Shimadzu and its number is A622 (https://www.shimadzu.com/an/sites/shimadzu.com.an/files/pim/pim_document_file/applications/application_note/13131/jpa120020.pdf, accessed on 17 July 2025), which involves single-wavelength absorbance measurement.

To quantify color, the absorbance of beer samples was measured at 430 nm, with turbidity correction performed by measuring absorbance at 700 nm. Absorbance readings were converted to European Brewery Convention (EBC) units using appropriate conversion factor.

Bitterness determination was performed on the same Shimadzu UV-Vis spectrophotometer following the standard bitterness analysis method in ASBC Methods of Analysis (Beer-23A). The procedure involved the extraction of is-alpha-acids from degassed beer with iso-octane in an acidic medium, followed by the measurement of the extract’s absorbance at 275 nm. Bitterness (IBU) was calculated as 50 times the absorbance at 275 nm.

#### 2.4.2. Determination of Alcohol by Volume (ABV)

The alcohol content of beer samples was determined by refractometry, using a portable refractometer. Readings of °BRIX were taken before and after fermentation. °BRIX values were corrected for the analytical methods of the American Society of Brewing Chemists (ASBC), and the specific gravity (SG) was calculated using the following equation to obtain the original extract (OG) and final extract (FG): SG = (°Brix/(258.6 − ((°Brix/258.2) × 227.1))) + 1. The alcohol content was estimated from specific gravity values, using the formula ABV = (OG − FG) × 131.25. Alcohol content was expressed as percent alcohol by volume (% *v*/*v*) [21,22].

#### 2.4.3. Foam Stability Analysis

Beer foam stability was determined using the shaking test described by Kapp and Bamforth [23]. Aliquots of 5 mL of beer were manually shaken ten times in test tubes, and foam height was measured in duplicate every 10 s (0–5 min) and every minute (5–10 min). Data were used to construct graphs using Microsoft Office Excel software.

Stability was evaluated by foam decline (drainage), calculating the relative decline velocity (RDV) by comparing it with the sample exhibiting the highest RDV. RDV was calculated as Δh/Δt, where Δh is the variation in foam displacement and Δt is the variation in time.

### 2.5. Statistical Analysis

All experiments were performed in duplicate, and results are expressed as mean ± standard deviation. Statistical analyses were performed using RGui statistical software version 4.5.1. Differences between groups were determined using a two-way Analysis of Variance (ANOVA), followed by a post hoc multiple comparison test (Tukey’s Honestly Significant Difference—HSD) to identify specific statistically significant differences. A *p*-value of less than 0.05 (*p* < 0.05) was considered statistically significant.

## 3. Results and Discussion

### 3.1. Enzymatic Activity

Prior to evaluating the operational conditions, the concentration of the AN-PEP enzyme was selected based on the established literature and previous studies regarding its application in brewing. A concentration of 2% (*v*/*v*) of the commercial enzyme solution, demonstrated as efficient in similar applications for gluten hydrolysis in beer [12,24] was utilized in all subsequent experiments. While the azocasein-based assay provides a general measure of proteolytic activity, it is important to acknowledge that it may not fully capture the enzyme’s specific activity towards proline-rich gluten peptides. More targeted and informative methods, such as those employing specific synthetic substrates (e.g., Z-Gly-Pro-pNA) or actual gluten peptides, have been proposed in the literature to assess AN-PEP activity with greater relevance to gluten degradation [25]. Despite this, the azocasein assay remains a widely accepted and practical method for general proteolytic activity screening in many food science applications.

**Figure 1:**
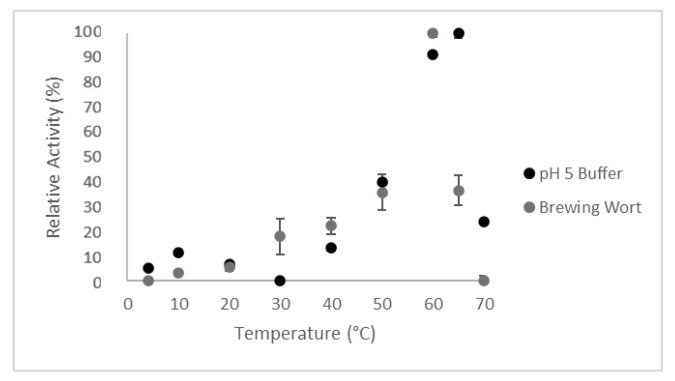
Effect of temperature on enzymatic activity in pH 5.0 buffer and brewing wort. Values are presented as mean ± standard deviation (SD) of 3 independent replicates (*n* = 3).

The influence of temperature on AN-PEP activity was investigated in two different media: pH 5.0 buffer and brewing wort, with temperatures ranging from 4 to 70 °C (Figure 1). The results demonstrated that enzymatic activity was significantly influenced by both temperature and the medium. In the pH 5.0 buffer, maximum activity was observed at 65 °C, whereas in brewing wort, the optimal temperature was slightly lower, peaking at 60 °C.

Figure 1 illustrates the effect of temperature on relative activity in pH 5.0 buffer and brewing wort. The relative activity values presented are normalized against the highest enzymatic activity observed for each respective medium: 60 °C in brewing wort and 65 °C in the pH 5.0 buffer. The difference in optimal temperatures between the two media can be attributed to the more complex nature of the brewing wort matrix, which, unlike the simplified buffer, contains a diverse array of biomolecules such as residual proteins, peptides, lipids, and other macromolecules. These components can interact with the enzyme, influencing its conformational flexibility, stability, and ultimately its optimal activity temperature. These results align with expectations for proteases that act in fermentation processes, where moderate temperatures favor catalysis [26,27]. Enzymatic activity at temperatures below and above the optimal range showed a decline, indicating the enzyme sensitivity to extreme temperature conditions, which can lead to a reduction in reaction rate.

**Figure 2:**
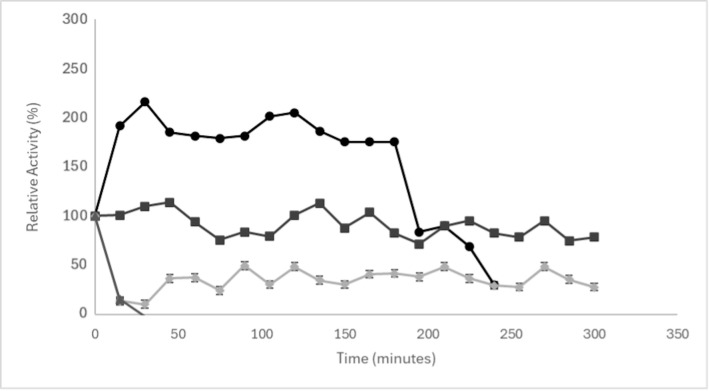
Thermal inactivation of AN-PEP enzyme at different temperatures: (●) 50 °C, (■) 65 °C, (◇) 75 °C, (▲) 100 °C. Values are presented as mean ± standard deviation (SD) of 3 independent replicates (*n* = 3).

To understand the stability of the AN-PEP enzyme under high thermal conditions, relevant for the brewing mashing and boiling processes, thermal inactivation experiments were conducted at four distinct temperatures: 50 °C, 65 °C, 75 °C, and 100 °C (Figure 2). The results indicate that enzyme inactivation is dependent on both temperature and exposure time.

It was observed that at 100 °C, AN-PEP inactivation was more rapid and pronounced, with a significant loss of activity within short periods, as expected for wort boiling temperature. At 75 °C, although inactivation was evident, the enzyme demonstrated greater stability compared to 100 °C. Regarding the lower temperatures, at 65 °C, the enzyme maintained a significant proportion of its activity for prolonged periods, being the temperature at which activity persisted longest among those tested. Notably, at 50 °C, the enzyme exhibited an apparent activation, where the measured relative activity increased to approximately twice its initial value, was maintained for a period, and then gradually declined towards the end of the experiment. Thermal inactivation is a critical point in the brewing process, as it allows control over the extent of hydrolysis and ensures the stability of the final product, preventing undesirable changes during storage. Rapid inactivation at 100 °C is desirable, as boiling aims, among other things, to inactivate enzymes to stabilize the wort and halt enzymatic reactions [19]. The maintenance of some activity at 65 °C for a prolonged period suggests that, although inactivation occurs, the enzyme can still contribute to hydrolysis during longer mashing phases, which can be beneficial depending on the specific objective of the process. This controlled activity is vital to prevent over-hydrolysis of proteins, which could negatively impact the final product characteristics, while also ensuring the stability and safety of the beer.

### 3.2. Optimization of Enzyme Application on the Beer Production Process

**Figure 3:**
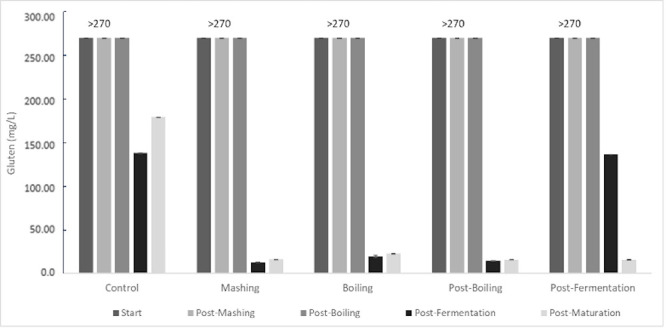
Gluten concentration (mg/L) at different production stages for the experimental beers. Bars represent the mean gluten concentration for each enzyme treatment (control, mashing, boiling, post-boiling, post-fermentation) across various production stages (start, post-mashing, post-boiling, post-fermentation, post-maturation). Values exceeding the quantification limit (LOQ) of the ELISA kit (>270 mg/L) are indicated numerically above the respective bars. Values are presented as mean ± standard deviation (SD) of 3 independent replicates (*n* = 3).

The effectiveness of AN-PEP enzyme addition in reducing gluten content was evaluated at different stages of the beer production process: at the beginning of mashing, at the beginning of boiling, after boiling, and after fermentation. A control beer, without enzyme addition, was also produced. Aliquots were collected at the beginning of the process (milled malt mixed with water), after mashing (30 min at 50 °C, 1 h at 65 °C, 10 min at 75 °C), after boiling (30 min at 100 °C), after fermentation (7 days at 20 °C), and after maturation (20 days at 4 °C). The results for gluten concentration are presented in Figure 3.

Initial results for all samples, including the control, showed gluten values above 270 mg/L, indicating that the gluten content was above the quantification limit (LOQ) of the ELISA kit employed at these stages, as numerically indicated above the bars in Figure 3. This phenomenon, where gluten reduction is not immediately apparent, is consistently observed in studies of enzyme-assisted beer production. The ELISA methodology, although robust, is designed to detect intact proteins or large antigenic gluten fragments [7,10]. During the initial mashing and boiling stages, the AN-PEP enzyme acts by hydrolyzing gluten into smaller peptides. However, many of these fragments may still be large enough to be recognized by the ELISA kit’s antibodies, resulting in high readings that do not reflect the true hydrolysis at a molecular level, but rather the presence of still-detectable antigenic peptides [11]. This is often attributed to antigen masking, where the complex matrix of the wort or beer in early stages may hinder the accessibility of gluten epitopes to the R5 antibody, or where partially hydrolyzed peptides still retain the specific epitope structure recognized by the antibody [11]. This challenge in early stage gluten quantification by ELISA is a recognized limitation across studies employing various proteolytic enzymes, as the generated peptide fragments may still retain some immunoreactivity despite significant hydrolysis [11]. For a more comprehensive understanding of gluten degradation kinetics, especially in complex matrices and early process stages, complementary analytical methods such as liquid chromatography coupled with mass spectrometry (LC-MS/MS) could provide a more targeted and precise quantification of specific gluten peptides [12,13,28].

It was only after the fermentation stage that detectable, and, for enzyme-treated samples, statistically significantly reduced gluten values were observed (*p* < 0.05). In beers where the enzyme was added at mashing, boiling, post-boiling, and post-fermentation, gluten values were 13.28 mg/L, 19.64 mg/L, 14.93 mg/L, and 136.5 mg/L, respectively. The most significant reduction was observed when the enzyme was added at mashing, boiling, and post-boiling, with gluten levels consistently below 20 mg/L (*p* < 0.05 compared to the control at the same stage). This high efficiency of AN-PEP in achieving ”gluten-free” thresholds is consistent with findings from other studies utilizing various proteolytic enzymes. For instance, while AN-PEP excels in broad-spectrum gluten hydrolysis, proteases from *A. oryzae* have also demonstrated significant gluten reduction, often with a focus on improving haze stability [15]. Similarly, enzymes from the *Bacillus* species, known for their diverse pH and temperature optima, have been explored, though their specific efficacy in achieving ”gluten-free” thresholds can vary depending on application conditions [16]. The observed rapid and substantial reduction by AN-PEP across different early addition points underscores its robust activity, aligning with its widespread industrial adoption compared to some other enzymatic alternatives that might require more specific conditions or combinations to reach similar efficacy [29].

The detection of a significant reduction in gluten after fermentation can be attributed to multiple factors. Firstly, the AN-PEP enzyme, primarily active during the mashing stages where optimal temperature conditions prevail, hydrolyzing gluten proteins into smaller peptides. While this enzymatic action occurs earlier in the process, the subsequent prolonged fermentation, characterized by lower temperatures compared to mashing, provides extended contact time for these hydrolyzed peptides to undergo further changes, such as aggregation, precipitation, and potential further enzymatic action or yeast-mediated modification. This leads to their removal from the liquid phase and, consequently, their non-recognition by the ELISA kit, which primarily detects larger antigenic fragments [11,30,31]. Furthermore, the presence of yeasts during fermentation may, in turn, indirectly influence enzymatic activity or peptide conformation, potentially contributing to the final degradation of peptides that were previously detectable [32]. The result of 136.5 mg/L for the “post-fermentation” addition was significantly higher than the other enzyme treatments (*p* < 0.05), indicating that when the enzyme is introduced so late in the process, it has less favorable time and conditions for substantial initial hydrolysis before the final analytical measurement. During maturation, gluten levels tended to stabilize or decrease slightly, consolidating the results obtained after fermentation.

**Figure 4:**
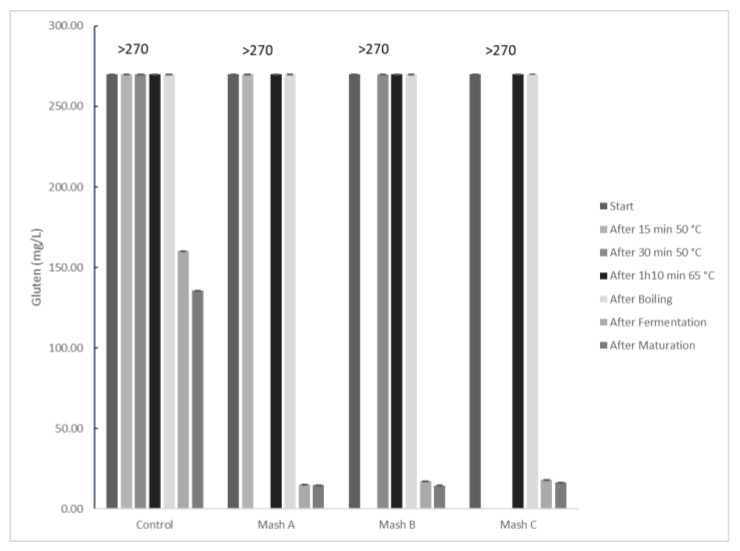
Gluten concentration (mg/kg) at different production stages for Control, Mash A, Mash B, and Mash C brewing processes. Values exceeding the quantification limit (LOQ) of the ELISA kit (>270 mg/kg) are indicated numerically above the respective bars. Values are presented as mean ± standard deviation (SD) of 3 independent replicates (*n* = 3).

Due to the results from the first stage, which indicated that enzyme addition during mashing was effective, additional experiments were conducted to investigate the impact of different mashing temperature ramps on gluten hydrolysis. A control beer and three experimental beers with enzyme added at the beginning of mashing were produced: Mash A (15 min at 50 °C), Mash B (30 min at 50 °C), and Mash C (beginning at 65 °C). Samples were collected at time zero, after 15 min, after 30 min, after 1 h and 10 min, after boiling, after fermentation, and after maturation. The results are presented in Figure 4.

Similar to previous experiments, gluten values in the initial stages for all samples remained above the quantification limit (270 mg/L). This reinforces the interpretation that, even with enzymatic activity occurring, the generated gluten fragments are still recognized by the ELISA kit in these early phases of the process. The complexity of the wort and the nature of gluten detection via immunoassay contributes to this apparent absence of immediate reduction. These trends are visually represented in Figure 4. Initial gluten concentrations were similar across all groups at the ”Start” stage. For the Control group, gluten levels remained relatively constant through the initial mashing steps (15 min 50 °C, 30 min 50 °C, 1 h 30 min 65 °C) and after boiling. In contrast, the Mash A, B, and C groups exhibited significant drops in gluten concentration much earlier in the process, reaching very low levels by the fermentation and maturation stages.

The comprehensive statistical analysis, specifically a two-way ANOVA, revealed significant main effects for mashing treatment (*p* < 0.0001) and production stage (*p* < 0.0001), as well as a significant interaction effect (*p* < 0.0001). This indicates that the gluten reduction profile is dependent on the specific process conditions and mashing treatment applied.

At the “After Fermentation” stage, Mash A, B, and C groups showed gluten contents of 15.19 mg/L, 17.2 mg/L, and 18.19 mg/L, respectively. Pairwise comparisons (Tukey’s HSD) at this stage indicated that the Control group had significantly higher gluten concentrations compared to all Mash A, Mash B, and Mash C groups (*p* < 0.0001 for all comparisons with Control). Importantly, statistical analysis revealed no significant differences in gluten content among Mash A, Mash B, and Mash C groups at the ”After Fermentation” stage (*p* > 0.05 for all pairwise comparisons among Mash A, B, and C). This suggests that, within the tested conditions, the specific duration of the 50 °C stage or its absence did not significantly impact the final gluten reduction, indicating the enzyme’s robustness under different mashing profiles. This finding is relevant for industrial optimization, as it suggests flexibility in the mashing profile without compromising the efficiency of gluten degradation by AN-PEP.

The results demonstrate that the enzyme is highly effective in hydrolyzing gluten proteins during beer production. The comprehensive statistical analysis confirmed significant effects of both mashing treatment and production stage, along with a crucial interaction effect, indicating that the gluten reduction profile is dependent on the specific process conditions. The non-detection of gluten reduction in early stages by ELISA, also observed in the statistical comparisons which showed no significant differences among groups at these initial points, followed by a drastic and statistically significant decrease after fermentation in the Mash groups (*p* < 0.0001 vs. Control), is a crucial finding. This highlights the complexity of gluten analysis in processed food matrices and the importance of considering the complete process. Enzymatic hydrolysis promoted by AN-PEP acts by breaking down immunogenic gluten peptides into smaller fragments. Although some of these fragments may still be detected by immunoassay-based methods (such as ELISA), the significant reduction observed after fermentation and maturation indicates that the enzyme is capable of cleaving the main toxic epitopes for celiac individuals into non-immunogenic sequences or sizes below the immunological detection limit [10,30,33]. Based on these findings, enzyme addition at the beginning of mashing is shown to be an efficient strategy to achieve gluten levels below regulatory “low-gluten” limits in the final product, regardless of the specific mashing temperature ramp tested. These findings contribute to the growing body of knowledge on enzymatic gluten degradation in beer, offering practical insights into optimizing AN-PEP application. While other enzymes show promise, AN-PEP’s consistent performance across varied mashing profiles, as demonstrated here, highlights its versatility and effectiveness in achieving regulatory gluten levels without compromising key quality parameters, positioning it as a leading choice for brewers aiming for low-gluten products.

### 3.3. Qualitative Beer Analysis

Color (EBC) and extract (°BRIX) were monitored at different stages of production for the control beer and for the experimental beers with the addition of the AN-PEP enzyme. Detailed results for each formulation are presented in Table 2 and Table 3.

In general, in all experiments, the expected pattern for beer production was observed. The extract (°BRIX) showed a sharp reduction after fermentation, reflecting the conversion of fermentable sugars into alcohol by yeast. Color (EBC) increased in the initial mashing and boiling stages due to the formation of melanoidins and other colored compounds [34].

Analyzing the enzyme-treated formulations, statistical evaluation revealed no significant differences in the behavior of °BRIX or color when compared to the control beer across the initial and intermediate stages of the process (e.g., initial point, after mashing, after boiling). This suggests that the presence of the enzyme, regardless of its addition time, did not significantly change after sugar extraction or the formation of color compounds during the hot stages of the process. Even in the later stages (fermentation and maturation), the observed °BRIX and EBC values for the enzyme-treated groups remained comparable to the control beer, with no statistically significant deviations.

Maintaining the color and °BRIX profiles close to the control is a positive result, as it indicates that the enzyme, despite its proteolytic hydrolysis function, does not negatively impact these basic sensory and physicochemical characteristics of beer that are important for product standardization.

**Figure 5:**
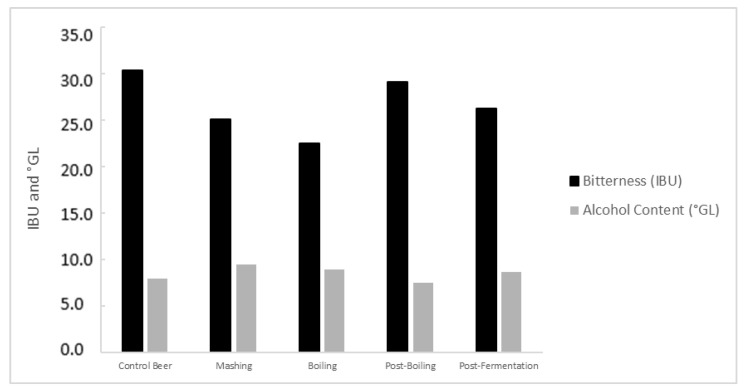
Analysis of bitterness (IBU) and alcohol content (°GL) as a function of enzyme addition stage. Values are presented as mean ± standard deviation (SD) of 3 independent replicates (*n* = 3).

Bitterness and alcohol content were determined in ready-to-consume beers (after maturation). The results are presented in Figure 5.

The results for bitterness showed that the control beer had an IBU of 30.4 ± 4.4 IBUs. Experimental beers with enzyme addition at the beginning of mashing, beginning of boiling, post-boiling, and post-fermentation showed IBU values of 25.1 ± 2.5 IBU, 22.5 ± 4.8 IBU, 29.2 ± 1.1 IBU, and 26.3 ± 2.2 IBU, respectively. Statistical analysis (one-way ANOVA) indicated no significant effect of enzyme addition stage on the final bitterness of the beers [F(4, 5) = 3.684, *p* = 0.0926]. While some numerical differences were observed, particularly for the boiling group, post hoc Tukey’s HSD tests confirmed that none of the pairwise comparisons between the control and enzyme-treated groups, or among the enzyme-treated groups, yielded statistically significant differences (all *p* > 0.05, e.g., Boiling vs. Control Beer, *p* = 0.0923). This outcome was anticipated, as AN-PEP is a prolyl endopeptidase with a specific action on proteins, thus not influencing the compounds primarily responsible for bitterness, such as hop iso-alpha-acids [35].

Regarding alcohol content, the control beer reached 8 ± 0.93 °GL. Beers with enzyme addition at mashing, beginning of boiling, post-boiling, and post-fermentation showed alcohol contents of 9.4 ± 0.20 °GL, 8.9 ± 0.20 °GL, 7.5 ± 2.78 °GL, and 8.7 ± 0.00 °GL, respectively. Statistical analysis (one-way ANOVA) revealed no significant effect of enzyme addition stage on alcohol content [F(4, 5) = 1.306, *p* = 0.381]. Similarly, post hoc Tukey’s HSD tests showed no statistically significant differences in alcohol content among any of the compared groups (all *p* > 0.05). Despite the lack of statistical significance, the numerical variations observed in alcohol content, such as slightly higher values for mashing and beginning of boiling additions (9.4 ± 0.20 °GL and 8.9 ± 0.20 °GL, respectively) and a slightly lower value for post-boiling addition (7.5 ± 2.78 °GL), warrant further discussion regarding their potential underlying mechanisms. These variations, even if not statistically significant in this specific experimental setup, could be indirectly associated with the action of the AN-PEP enzyme on wort proteins and their subsequent impact on yeast performance and fermentability. By hydrolyzing gluten proteins and other larger protein molecules in the wort, the enzyme may alter the profile of available nitrogenous compounds (e.g., free amino nitrogen—FAN) that are crucial nutrients for yeast growth and metabolism. While a certain level of protein hydrolysis is beneficial for providing FAN, excessive or altered protein breakdown could potentially lead to suboptimal nutrient availability or changes in wort viscosity, which might influence yeast activity and fermentation kinetics [36]. Alternatively, changes in the protein profile due to enzymatic action could affect yeast flocculation and sedimentation behavior. Proteins play a role in yeast aggregation, and their hydrolysis might alter the extent or timing of flocculation, potentially impacting the efficiency of sugar conversion and, consequently, the final alcohol yield [36,37,38]. Furthermore, the interaction between hydrolyzed peptides and yeast cell walls could influence nutrient uptake or stress responses, indirectly affecting fermentation performance. Considering these alcohol levels and the initial °Brix of the wort, these results suggest a potential for high gravity brewing in some experimental conditions. This approach, common in the brewing industry, involves producing a higher-alcohol wort that can later be diluted, optimizing fermentation tank utilization and increasing final production volume. The enzyme’s ability to maintain or slightly increase fermentability in certain early addition scenarios, even without statistically significant differences, suggests a beneficial interaction that could be further optimized for industrial applications where maximizing fermenter output is critical. However, it is important to note that the observed numerical variations are generally within acceptable ranges for most beer styles, indicating that the overall impact on alcohol content does not fundamentally compromise the product’s characteristics.

The qualitative beer analyses demonstrated that the application of the AN-PEP enzyme, under the tested conditions, did not cause undesirable changes in color, °BRIX, and bitterness parameters. Variations in alcohol content were observed but generally remained within acceptable limits for product style. These results, in conjunction with the effectiveness in gluten reduction, indicate that the AN-PEP enzyme is a promising tool for producing low-gluten beers that preserve the expected physicochemical and sensory characteristics.

### 3.4. Foam Stability

Foam stability is a critical quality attribute in beer, highly valued by consumers, and fundamentally influenced by the formation of a stable emulsion involving proteins, carbohydrates, and gases [35]. Proteins, particularly proline-rich polypeptides, play a crucial role in forming and stabilizing the protein network at the liquid–gas interface, which is essential for a robust foam structure [39,40]. When proteolytic enzymes are introduced, they can hydrolyze these foam-active proteins, potentially compromising foam formation and stability.

Foam stability was first evaluated in beers produced with AN-PEP enzyme addition at different stages of the process, comparing them with the control beer. The foam decline profiles over time for each sample are presented in Table 4. In this table, “Initial Height (mm)” refers to the foam height immediately after pouring, while ”Final Height (mm)” represents the foam height remaining after the ”Time to Foam Disappearance (s)” period indicated, or at the end of the 300 s test if the foam did not fully disappear within that timeframe.

The results demonstrate distinct foam behaviors. The control beer, along with beers that received enzyme addition at the beginning of boiling and after boiling exhibited the slowest and most linear foam decline profiles, with foam persisting throughout the 300 s test period. For instance, the Control beer retained 34 mm of its initial 47 mm foam height after 300 s, while the post-boiling beer retained 36 mm of its initial 49 mm foam height. These profiles indicate high foam stability. In contrast, samples with enzyme addition at mashing and, especially, after fermentation showed a significantly faster foam decay. For the mashing and boiling additions, foam completely disappeared within 100 s, and for post-fermentation, foam disappeared within 120 s, indicating a substantial reduction in foam stability.

Beer foam stability is a complex attribute, influenced by high molecular weight proteins, such as barley proteins (prolamins and glutelins), carbohydrates, polyphenols, and hop oils [35]. The observed reduction in foam stability when the AN-PEP enzyme was added during mashing and after fermentation is an expected result and consistent with the literature for proteolytic enzymes. AN-PEP, being a prolyl endopeptidase, acts by hydrolyzing proline-rich proteins, which are crucial components for foam formation and stabilization [39,40]. The breakdown of these proteins into lower molecular weight peptides compromises the foam structure, which depends on the formation of a stable protein network at the liquid–gas interface [41]. Excessive hydrolysis of these proteins results in peptides that are either too small or have an altered conformation, rendering them unable to effectively migrate to the air–liquid interface or form the necessary stable viscoelastic film, leading to a faster collapse.

The maintenance of foam stability in samples with enzyme addition at the beginning of boiling and after boiling, however, is an important finding. This can be primarily attributed to the thermal inactivation of the AN-PEP enzyme’s activity and conformation. For the enzyme added at the beginning of boiling, rapid denaturation occurs due to the high temperatures (100 °C) of the boiling process (Figure 2). This immediate inactivation significantly reduces the enzyme’s capacity to hydrolyze foam-forming proteins, thereby minimizing its negative impact on this attribute. Similarly, for the addition after boiling, while the enzyme itself was not subjected to boiling-induced inactivation, its activity during subsequent steps (e.g., fermentation at 20 °C and maturation at 4 °C) would be significantly reduced due to suboptimal lower temperatures. This decreased activity at colder temperatures, combined with proteins potentially already activated during previous hot-side processes, contributes to the preservation foam-forming capacity. On the other hand, during mashing, where the enzyme remains active for a longer period and under optimal hydrolysis conditions (Figure 1), it leads to a more pronounced degradation of foam proteins. The enzyme’s sustained activity under these conditions results in the cleavage of foam-active proteins into peptides that are too small or lack the appropriate amphiphilic balance to contribute effectively to foam stability. For addition after fermentation, although the temperature is lower, the enzyme may still exert a direct effect on the protein network already present in the finished beer. In this scenario, the proteins crucial for foam stability might be more vulnerable as they have passed through all previous processing steps, and there is insufficient time or conditions for restructuring of the protein matrix in the final beer, leading to a faster collapse.

These findings have significant implications for practical brewing scenarios and highlight crucial formulation trade-offs in industrial applications aiming for low-gluten beers. Firstly, while enzyme addition during mashing is highly effective in achieving rapid and substantial gluten reduction (as shown in Figure 3), it comes at a significant cost to foam stability. For brewers, this presents a clear trade-off: prioritizing maximum gluten reduction via mashing addition may necessitate the use of alternative foam-enhancing ingredients (e.g., specific hop products, specialized malt, or adjuncts) or process adjustments (e.g., nitrogenating, optimized carbonation) to compensate for the proteolytic degradation of foam-active proteins. Consumer acceptance is heavily influenced by foam quality, making this a critical consideration for market viability. Secondly, the preservation of foam stability with enzyme addition at the beginning of boiling or after boiling offers a valuable alternative for brewers. Although the gluten reduction might be slightly less pronounced compared to mashing addition, it still achieves ”low-gluten” levels while maintaining a desirable foam profile. This suggests that for industrial applications, the boiling or post-boiling addition points represent a more balanced approach, allowing for gluten reduction without compromising a key quality attribute. This strategy minimizes the need for additional foam-stabilizing agents, simplifying the formulation, and potentially reducing production costs. In essence, the choice of enzyme addition point is a strategic decision that depends on the specific priorities of the brewer. If the absolute lowest gluten content is paramount, mashing addition is effective but requires compensatory measures for foam. If a balance between low gluten and acceptable foam quality is desired, boiling or post-boiling additions are more suitable. This research provides clear guidance for brewers to make informed decisions regarding AN-PEP application based on their product specifications and desired quality profile.

The qualitative beer analyses demonstrated that the application of the AN-PEP enzyme under the tested conditions allowed the production of low-gluten beers without causing undesirable changes in color and bitterness parameters. A slight variation in alcohol content was observed, but it remained within acceptable limits, though statistical analysis indicated no significant differences. However, foam stability was significantly reduced when the enzyme was added during mashing or after fermentation, due to the hydrolysis of foam-forming proteins. In contrast, enzyme addition at the beginning of boiling or after boiling preserved foam stability, probably due to the thermal denaturation of AN-PEP at these stages. These findings highlight the critical importance of the enzyme’s addition point in the process to optimize both gluten reduction and the maintenance of essential quality attributes, such as foam stability, for the final beer quality.

## 4. Conclusions

This study successfully demonstrated the efficacy of the AN-PEP enzyme in producing low-gluten beers while evaluating its impact on key quality attributes. The AN-PEP enzyme proved highly effective in reducing gluten content to regulatory “low-gluten” levels, with additions at the beginning of mashing and boiling yielding the most efficient results.

Regarding beer quality, the application of AN-PEP did not significantly compromise color, extract (°BRIX), bitterness, or alcohol content. However, foam stability was significantly affected when the enzyme was added during mashing or after fermentation, attributed to the hydrolysis of foam-forming proteins. Conversely, enzyme addition at the beginning of boiling or after boiling largely preserved foam stability, likely due to the high temperatures of the boiling process denaturing the enzyme, thus limiting its activity on foam-positive proteins. When added after boiling, the lower temperatures of the wort after cooling would naturally reduce the enzyme’s overall activity, thereby minimizing its impact on foam stability.

In conclusion, the AN-PEP enzyme is a promising tool for producing low-gluten beers. Our findings highlight the critical importance of the enzyme’s addition point: for effective gluten reduction with minimal impact on essential physicochemical and sensory characteristics, particularly foam stability, addition at the beginning of boiling or after boiling emerged as the most promising strategies. These results offer valuable insights that can be readily scaled to pilot or industrial brewing systems, paving the way for broader adoption of AN-PEP in low-gluten beer production. Future research is recommended to optimize enzyme dosage and conduct comprehensive sensory evaluations for consumer acceptance, ideally incorporating a diverse consumer panel to further validate the sensory profile of AN-PEP treated beers. We hope that our results can help brewers to produce gluten-free beer with improved physicochemical characteristics, such as foam stability, color, bitterness, and alcohol content, thereby reconciling food safety and quality attributes.

## Figures and Tables

**Table 1:** Summary of experimental conditions for AN-PEP enzyme application in beer production.

Experiment Series	Enzyme Addition Point	Temperature Profile/Mashing Ramp	Estimated Enzyme Exposure Time /Conditions
Control	-	Standard (50 °C/30 min, 65 °C/1 h, 75 °C/10 min)	N/A
Addition Point Series		Standard (50 °C/30 min, 65 °C/1 h, 75 °C/10 min)	
	Beginning of Mashing	50 °C	Throughout mashing (up to 75 °C inactivation)
	Beginning of Boiling	100 °C	During boiling (rapid inactivation)
	Post-Boiling	20 °C	During fermentation and maturation
	Post-Fermentation	4 °C	During maturation
Mashing Ramp Series	Beginning of Mashing		Throughout mashing (up to 75 °C inactivation)
	Mash A	50 °C/15 min, 65 °C/1 h, 75 °C/10 min	
	Mash B	50 °C/30 min, 65 °C/1 h, 75 °C/10 min	
	Mash C	65 °C/1 h, 75 °C/10 min (no 50 °C rest)	

**Table 2:** Variation of °BRIX values throughout production stages for experimental beers. Values are presented as mean ± standard deviation (SD) of 3 independent replicates (*n* = 3). Statistical analysis (two-way ANOVA) revealed no statistically significant differences (*p* > 0.05) in °BRIX values between enzyme-treated and control beers across all production stages.

Brewing Step of Enzyme Addition	Initial Point	After Mashing	After Boiling	After Fermentation	After Maturation
Control	2.8 ± 0.5	9.0 ± 5,6	13.1 ± 1.5	7 ± 0.8	7.2 ± 1.3
Mashing	2.8 ± 0.5	12.1 ± 4.1	16.8 ± 0.8	9.1 ± 0.7	9.6 ± 0.5
Boiling	2.8 ± 0.5	9.9 ± 1.8	16.7 ± 0.4	9.6 ± 0.5	9.9 ± 0.1
Post-Boiling	2.8 ± 0.5	9.9 ± 1.1	14.7 ± 1.4	9.0 ± 0.7	9.5 ± 0.1
Post-Fermentation	2.8 ± 0.5	11.1 ± 2.6	13.7 ± 0.1	7.1 ± 0.1	9.7 ± 0.1

**Table 3:** Variation of EBC values throughout production stages for experimental beers. Values are presented as mean ± standard deviation (SD) of 3 independent replicates (*n* = 3). Statistical analysis (two-way ANOVA) revealed no statistically significant differences (*p* > 0.05) in or EBC values between enzyme-treated and control beers across all production stages.

Brewing Step of Enzyme Addition	Initial Point	After Mashing	After Boiling	After Fermentation	After Maturation
Control	54.5 ± 1.5	55.5 ± 1.5	75.8 ± 1.5	16.5 ± 1.7	13.2 ± 0.4
Mashing	54.5 ± 1.5	62.8 ± 5.5	85.6 ± 2.8	5.8 ± 0.7	8.3 ± 0.8
Boiling	54.5 ± 1.5	42.2 ± 3.3	66.1 ± 2.8	5.4 ± 0.08	7.8 ± 0.8
Post-Boiling	54.5 ± 1.5	48.4 ± 0.01	53.0 ± 0.2	13.2 ± 0.005	12.3 ± 0.008
Post-Fermentation	54.5 ± 1.5	48.4 ± 0.1	53.0 ± 0.2	13.2 ± 0.1	12.3 ± 0.08

**Table 4:** Foam stability of experimental beers with enzyme addition at different process stages. Values are presented as mean ± standard deviation (SD) of 3 independent replicates (*n* = 3). Statistical analysis (one-way ANOVA followed by Tukey’s HSD post hoc test) was performed, revealing statistically significant differences in foam stability between certain enzyme treatments and the control, as discussed in the text.

Sample	Initial Foam Height (mm)	Final Foam Height (mm)	Time to Foam Disappearance (s)
Control	47	34	>300
Mashing	42	27	100
Boiling	40	31	100
Post-Boiling	49	36	>300
Post-Fermentation	46	33	120

## Data Availability

The original contributions presented in the study are included in the article, further inquiries can be directed to the corresponding author.

## References

[1] Fernandes R., Borges L.A., Anacleto D., de Souza M.S.N., da Silva L.B.M., Monroy Y.M., Batista E.A.C., Machado A.P.O. (2025). Gluten-free cookies: A comprehensive review of substitutes for wheat flour. Food Humanit..

[2] Lebwohl B., Rubio-Tapia A. (2021). Epidemiology, Presentation, and Diagnosis of Celiac Disease. Gastroenterology.

[3] Scherf K.A., Wieser H., Koehler P. (2018). Novel approaches for enzymatic gluten degradation to create high-quality gluten-free products. Food Res. Int..

[4] Meussdoerffer F.G. (2009). A Comprehensive History of Beer Brewing. Handbook of Brewing: Processes, Technology, Markets.

[5] Rubio-Flores M., Serna-Saldivar S.O. (2016). Technological and Engineering Trends for Production of Gluten-Free Beers. Food Eng. Rev..

[6] Cela N., Condelli N., Caruso M.C., Perretti G., Di Cairano M., Tolve R., Galgano F. (2020). Gluten-free brewing: Issues and perspectives. Fermentation.

[7] Partichelli C.P., Silveira V.C.C., Manfroi V., Rodrigues R.C. (2024). Exogenous enzymes for gluten-free beer production: A review of the industrial practice and its implications for scientific research. Innov. Food Sci. Emerg. Technol..

[8] Standards|CODEX ALIMENTARIUS FAO-WHO. https://www.fao.org/fao-who-codexalimentarius/codex-texts/list-standards/en/.

[9] Panda R., Garber E.A.E. (2019). Detection and quantitation of gluten in fermented-hydrolyzed foods by antibody-based methods: Challenges, progress, and a potential path forward. Front. Nutr..

[10] Amnuaycheewa P., Niemann L., Goodman R.E., Baumert J.L., Taylor S.L. (2022). Challenges in Gluten Analysis: A Comparison of Four Commercial Sandwich ELISA Kits. Foods.

[11] Lacorn M., Weiss T. (2015). Partially Hydrolyzed Gluten in Fermented Cereal-Based Products by R5 Competitive ELISA: Collaborative Study, First Action 2015.05. J. AOAC Int..

[12] Fiedler K.L., Cao W., Zhang L., Naziemiec M., Bedford B., Yin L., Smith N., Arbuckle M., Lopez-Hernandez A., Jackson L.S. (2019). Detection of gluten in a pilot-scale barley-based beer produced with and without a prolyl endopeptidase enzyme. Food Addit. Contam. Part A.

[13] Melini F., Melini V. (2018). Immunological Methods in Gluten Risk Analysis: A Snapshot. Safety.

[14] Fernández-Gil M.D.P., Simon E., Gibert A., Miranda J., Roger Alcoba E., Vilchez Cerezo E., Martínez O., Bustamante M.Á. (2021). Gluten assessment in beers: Comparison by different commercial ELISA kits and evaluation of NIR analysis as a complementary technique. Foods.

[15] Kang C., Yu X.W., Xu Y. (2015). Cloning and expression of a novel prolyl endopeptidase from *Aspergillus oryzae* and its application in beer stabilization. J. Ind. Microbiol. Biotechnol..

[16] Wang J., Xu A., Wan Y., Li Q. (2013). Purification and characterization of a new metallo-neutral protease for beer brewing from *Bacillus amyloliquefaciens* SYB-001. Appl. Biochem. Biotechnol..

[17] Akeroyd M., van Zandycke S., den Hartog J., Mutsaers J., Edens L., van den Berg M., Christis C. (2016). AN-PEP, proline-specific endopeptidase, degrades all known immunostimulatory gluten peptides in beer made from barley malt. J. Am. Soc. Brew. Chem..

[18] Guerdrum L.J., Bamforth C.W. (2012). Prolamin levels through brewing and the impact of prolyl endoproteinase. J. Am. Soc. Brew. Chem..

[19] Di Ghionno L., Marconi O., Sileoni V., De Francesco G., Perretti G. (2017). Brewing with prolyl endopeptidase from *Aspergillus niger*: The impact of enzymatic treatment on gluten levels, quality attributes and sensory profile. Int. J. Food Sci. Technol..

[20] Pessato T.B., de Carvalho N.C., Tavano O.L., Fernandes L.G.R., Zollner R.d.L., Netto F.M. (2016). Whey protein isolate hydrolysates obtained with free and immobilized Alcalase: Characterization and detection of residual allergens. Food Res. Int..

[21] Papazian C. (2014). The Complete Joy of Homebrewing Fourth Edition: Fully Revised and Updated.

[22] Cutaia A.J., Reid A.J., Speers R.A. (2009). Examination of the Relationships Between Original, Real and Apparent Extracts, and Alcohol in Pilot Plant and Commercially Produced Beers. J. Inst. Brew..

[23] Kapp G.R., Bamforth C.W. (2002). The foaming properties of proteins isolated from barley. J. Sci. Food Agric..

[24] Walter T., Wieser H., Koehler P. (2014). Production of gluten-free wheat starch by peptidase treatment. J. Cereal Sci..

[25] Benucci I., Caso M.C., Bavaro T., Masci S., Keršienė M., Esti M. (2020). Prolyl endopeptidase from *Aspergillus niger* immobilized on a food-grade carrier for the production of gluten-reduced beer. Food Control.

[26] Kubota K., Tanokura M., Takahashi K. (2005). Purification and characterization of a novel prolyl endopeptidase from *Aspergillus niger*. Proc. Jpn. Acad. Ser. B.

[27] Tavano O.L. (2013). Protein hydrolysis using proteases: An important tool for food biotechnology. J. Mol. Catal. B Enzym..

[28] Henrottin J., Planque M., Huet A.-C., Marega R., Lamote A., Gillard N. (2019). Gluten Analysis in Processed Foodstuffs by a Multi-Allergens and Grain-Specific UHPLC-MS/MS Method: One Method to Detect Them All. J. AOAC Int..

[29] Kerpes R., Fischer S., Becker T. (2017). The production of gluten-free beer: Degradation of hordeins during malting and brewing and the application of modern process technology focusing on endogenous malt peptidases. Trends Food Sci. Technol..

[30] Diaz-Amigo C., Popping B. (2013). Accuracy of ELISA detection methods for gluten and reference materials: A realistic assessment. J. Agric. Food Chem..

[31] Lexhaller B., Tompos C., Scherf K.A. (2016). Comparative analysis of prolamin and glutelin fractions from wheat, rye, and barley with five sandwich ELISA test kits. Anal. Bioanal. Chem..

[32] Liu C.J., Chopra A.K., Strnadová M., Chaloupka J. (1984). Degradation of abnormal proteins in growing yeast. FEMS Microbiol. Lett..

[33] Cebolla Á., Moreno M.d.L., Coto L., Sousa C. (2018). Gluten Immunogenic Peptides as Standard for the Evaluation of Potential Harmful Prolamin Content in Food and Human Specimen. Nutrients.

[34] Garcia-Moreno H., Calvo J.R., Maldonado M.D. (2013). High levels of melatonin generated during the brewing process. J. Pineal Res..

[35] Bamforth C.W. (2017). Progress in brewing science and beer production. Annu. Rev. Chem. Biomol. Eng..

[36] Ledley A.J., Elias R.J., Cockburn D.W. (2023). Evaluating the Role of Mashing in the Amino Acid Profiles of Worts Produced from Gluten-Free Malts. Beverages.

[37] Krogerus K., Gibson B.R. (2013). 125th anniversary review: Diacetyl and its control during brewery fermentation. J. Inst. Brew..

[38] Maicas S. (2020). The Role of Yeasts in Fermentation Processes. Microorganisms.

[39] Bamforth C.W. (1985). The foaming properties of beer. J. Inst. Brew..

[40] Bamforth C.W. (2023). The physics and chemistry of beer foam: A review. Eur. Food Res. Technol..

[41] Bamforth C.W. (2004). The relative significance of physics and chemistry for beer foam excellence: Theory and practice. J. Inst. Brew..

